# The fallacy of enzymatic hydrolysis for the determination of bioactive curcumin in plasma samples as an indication of bioavailability: a comparative study

**DOI:** 10.1186/s12906-019-2699-x

**Published:** 2019-11-04

**Authors:** Sidney J. Stohs, C. Y. O. Chen, Harry G. Preuss, Sidhartha D. Ray, Luke R. Bucci, Jin Ji, Kevin J. Ruff

**Affiliations:** 1 School of Pharmacy and Health Professions, Creighton University Medical Center, Omaha, NE USA; 2 Biofortis Research, Addison, IL USA; 3 Department of Biochemistry, Georgetown University Medical Center, Washington, DC USA; 4 Department of Pharmaceutical and Biomedical Sciences, Touro College of Pharmacy, Manhattan, NY USA; 5 Interpath Nutrition, Reno, NV USA; 6 PulchriBio Intl, Cambridge, MA USA; 7 Stratum Nutrition, Carthage, MO USA

**Keywords:** Bioactive curcumin, Curcumin glucuronide, Curcumin sulfate, Enzymatic hydrolysis

## Abstract

**Background:**

Numerous health benefits have been demonstrated for curcumin which is extracted from turmeric (*Curcuma longa* L). However, due to its poor absorption in the free form in the gastrointestinal tract and rapid biotransformation, various formulations have been developed to enhance its bioavailability. Previous studies indicate that the free form of curcumin is more bioactive than its conjugated counterparts in target tissues. Most curcumin pharmacokinetics studies in humans designed to assess its absorption and bioavailability have measured and reported total (free plus conjugated) curcumin, but not free, bioactive curcumin in the plasma because enzymatic hydrolysis was employed prior to its extraction and analysis. Therefore, the bioavailability of free curcumin cannot be determined.

**Methods:**

Eight human subjects (4 male, 4 female) consumed a single dose of 400 mg curcumin in an enhanced absorption formulation, and blood samples were collected over 6 h. Plasma was treated either with or without glucuronidase/sulfatase prior to extraction. Curcumin and its major metabolites were analyzed using HPLC-tandem mass spectrometry. In addition, the literature was searched for pharmacokinetic studies involving curcumin using PubMed and Google Scholar, and the reported bioavailability data were compared based on whether hydrolysis of plasma samples was used prior to sample analysis.

**Results:**

Hydrolysis of blood plasma samples prior to extraction and reporting the results as “curcumin” obscures the amount of free, bioactive curcumin and total curcuminoids as compared to non-hydrolyzed samples. As a consequence, the data and biological effects reported by most pharmacokinetic studies are not a clear indication of enhanced plasma levels of free bioactive curcumin due to product formulations, leading to a misrepresentation of the results of the studies and the products when enzymatic hydrolysis is employed.

**Conclusions:**

When enzymatic hydrolysis is employed as is the case with most studies involving curcumin products, the amount of free bioactive curcumin is unknown and cannot be determined. Therefore, extreme caution is warranted in interpreting published analytical results from biological samples involving ingestion of curcumin-containing products.

**Trial registration:**

ClinicalTrails.gov, trial identifying number NCT04103788, September 24, 2019. Retrospectively registered.

## Background

Curcumin is the active polyphenolic constituent in turmeric derived from the rhizomes of *Curcuma longa* L. Numerous human, animal and in vitro studies have demonstrated the benefits of curcumin in health promotion and prevention via an array of bio-actions, including antioxidant, anti-inflammatory, cytoprotective, immuno-modulating, metabolism regulating, antibacterial, anti-fungal, antiviral, antineoplastic, and anti-depressant properties [1–11]. However, unformulated and unprocessed [regular] curcumin is highly insoluble in water, and is known for its poor gastrointestinal absorption and bioavailability. This limits its biological and physiological effects at target tissues, leading to restricted usefulness in general healthcare and disease prevention. To address this issue, various formulations have been developed to facilitate the bioavailability of curcumin [12, 13].

The current study assessed the effects of enzymatic hydrolysis vs direct plasma extraction without hydrolysis on the plasma levels of curcumin and its metabolites. The bioavailability of commercially available curcumin formulations was also compared and contrasted based on the methods used in the preparation of plasma samples for assessing the relative and comparative absorption of curcumin. The literature was searched for pharmacokinetics studies involving curcumin in PubMed and Google Scholar. Bioavailability is measured by calculating the area under the curve (AUC) of the concentration time profile of a substance as curcumin. The rate of absorption was expressed as the maximum concentration (C_max_) and time (t_max_) at which the maximum concentration is reached. By general definition, bioequivalence can be defined as the absence of significant differences between different products or formulations in the rate and extent [bioavailability] to which an active ingredient becomes available to the site of action when administered at the same molar dose under similar conditions such that both safety and efficacy are the same [14].

### Curcumin metabolism

**Fig. 1:**
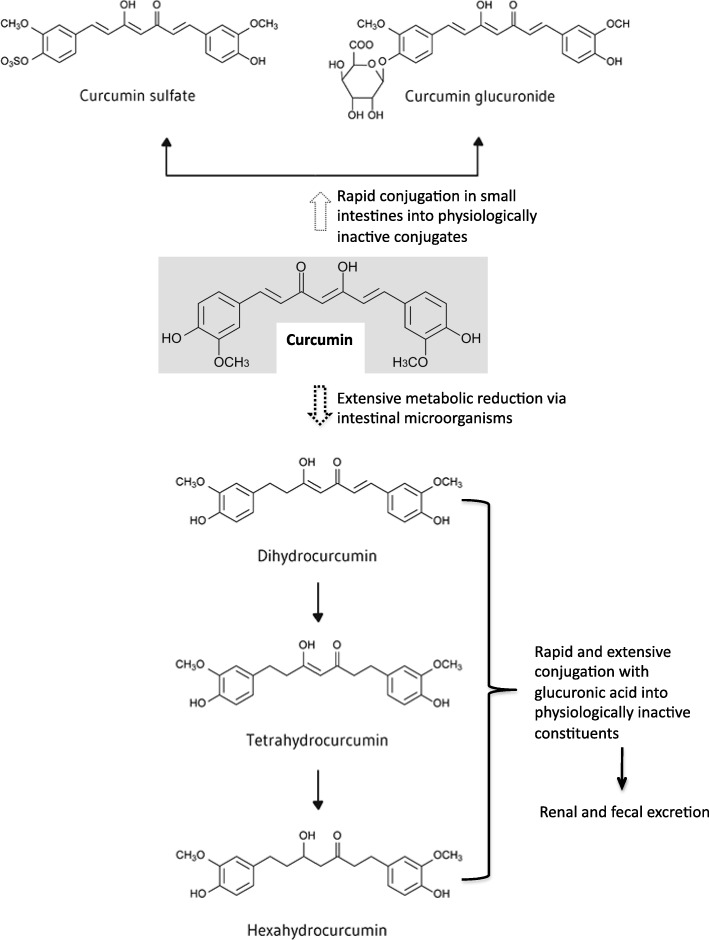
Metabolic Pathways of Curcumin

Curcumin when consumed orally undergoes rapid conjugation in the small intestine, liver and kidneys to curcumin glucuronide, curcumin sulfate and methylated curcumins which undergo rapid excretion in the urine and feces [4, 5, 7, 11–16]. The primary metabolic pathways for curcumin are presented in Fig. 1. Curcumin occurs in the blood primarily as these physiologically and pharmacologically inactive conjugates with relatively little free, bioactive curcumin, which is similar to other polypyhenols. Extensive metabolic reduction to dihydrocurcumin, tetrahydrocurcumin and hexahydrocurcumin also occurs via intestinal microorganisms [5, 11–18] (Fig. 1). All of these reduction products may have physiological activities. However, these metabolites also undergo rapid and extensive conjugation with glucuronic acid, thus converting them into physiologically inactive constituents which are eliminated via renal and fecal excretion [5, 11–13, 15–18]. Similar metabolic pathways exist for the minor curcuminoids demethoxycurcumin and bis-demethoxycurcumin.

Since curcumin is more physiologically active as compared to its conjugated forms, it is generally assumed that blood levels of free curcumin reflect its bio-efficacy [5, 11–13, 15–18]. It is not clear what form of curcumin exists within tissues and what constitutes the active form at the cellular and molecular levels. Studies have suggested that various oxidation products of curcumin may be responsible for at least some of the biological activities [19–21]. Furthermore, it is not known whether conjugated forms of curcumin as curcumin glucuronide reach target tissues and upon dissociation free curcumin is released.

Human data on other polyphenols, such as quercetin, have found small amounts of quercetin glucuronide in macrophages of injured/inflamed sites of the human brain [22]. Macrophages possess glucuronidase activity, raising the possibility of polyphenol deconjugation, but at only specific, diseased locations [22]. Deconjugation of curcumin glucuronide under similar circumstances has not been determined. Free curcumin in the blood (plasma) currently is the best indicator of bioavailability and bioequivalence.

### Curcumin formulations

Various approaches have been used to overcome the poor absorption, rapid phase 2 metabolism, and poor bioavailability of curcumin [12, 13, 22, 23]. These strategies include formulations with micelles, liposomes or interaction with macromolecules such as gelatin, and various polysaccharides [12]. In addition, nano-particulate preparations of curcumin to enhance bioavailability have been developed including nano-micelles, nano-emulsions, nano-gels, polymers, dendrimers, conjugates and solid dispersions. Although these formulations have demonstrated varying degrees of increased absorbability of total curcumin, some of these formulations have limited applications due to non-food grade ingredients, large material loads with small curcumin delivery loads, or various regulatory issues [12].

Various formulations that have been developed include: a liquid droplet nanomicellar formulation containing Gelucire® and polysorbate 20 (BioCurc®); micronized curcuminoids plus turmeric oil (BCM-95®; BioCurcumax®); co-administered with piperine (Curcumin C^3^ Complex®); formulated with phosphatidylcholine from soy lecithin and microcrystalline cellulose (Meriva®); a solid lipid curcumin particle (Longvida®); complexed with a hydrophobic carrier, cellulosic derivatives and natural antioxidants (CurcuWIN®); a micro-particle surface-controlled colloidal dispersion using ghatti gum and glycerin (Theracurmin®); complexed with fenugreek-derived galactomannan fiber (CurQfen®); complexed with γ-cyclodextrin (Cavacurmin®); a matrix consisting of glycerol esters of fatty acids, medium chain triglycerides, hydroxymethylcellulose, sodium alginate and microcrystalline cellulose (MicroActive Curcumin); a mixture of surfactants, polar lipids and solvents (Hydrocurc™), a complex of triacetin and panodan spray-dried on porous silicon dioxide (Micronized Curcumin); a whey-protein-curcumin conjugate (CurcuminPro®), and a natural turmeric matrix formulation composed of carbohydrates, proteins, fiber and volatile oil (Acumin®/Cureit®) [12, 13, 24–38].

Pharmacokinetic studies have been conducted with various curcumin formulations. However, it is very difficult to compare and contrast the results from these formulations because numerous experimental factors may influence the results in addition to the inherent effects of the formulations. Among the factors that influence the results are the actual dose and dosage form (tablet, capsule, softgel, and liquid) of curcumin, manner of administration (with water or food or empty stomach), subject characteristics and demographics (gender, ethnicity, age, weight, diet, and others), plasma collection time points and duration, and analytical methods (extraction, enzymatic hydrolysis, assay and detection). In addition, some studies have not provided adequate experimental detail to ascertain what was actually measured. Furthermore, it is not always clear whether the term “curcumin” refers to actual curcumin or to a group of curcuminoids. As a consequence, it is very difficult and may be inaccurate to pool data from the various studies for comparative purposes.

The most appropriate approach to comparing products is a direct head to head pharmacokinetic comparison in a cross-over designed study with normalization of the results on the basis of C_max_/mg curcumin and AUC/mg curcumin administered. The C_max_ is the maximum (peak) concentration of a substance (curcumin), and area under the curve (AUC) provides information on the amount (extent) of curcumin absorbed over a finite period of time. The major pharmacokinetic index used for determining bioequivalence (rate and extent of absorption) of various curcumin products is a graphic plot of blood plasma concentration of the active constituent(s) against time (AUC) [14, 39, 40].

Although the C_max_ is related to absorbability, it does not reveal how much of the curcumin was absorbed. AUC provides a direct measurement of bioavailability [14, 39, 40]. Therefore, when comparing bioavailability, the AUC/mg derived from normalization with the administered dose provides a means of direct comparison. Unfortunately, claims of enhanced absorbability are sometimes made in marketing materials based on only the C_max_ values between a given product and unformulated standard 95% curcumin or with other formulations of curcumin.

### Hydrolysis vs no hydrolysis

A major pitfall of most pharmacokinetic studies involving the diverse curcumin formulations has been the lack of demonstrated increase in free, bioactive curcumin in the blood [26–38]. With relatively few exceptions [13, 24, 26], plasma samples are routinely subjected to hydrolysis with the enzymes β-glucuronidase and sulfatase most commonly from *Helix pomatia* snail extracts to generate total curcumin [26–38] because curcumin glucuronide and curcumin sulfate are the predominant circulating but physiologically inactive conjugates of curcumin. Thus, the resulting conclusions do not provide a clear understanding of the potential pharmacokinetic benefits of the formulations with respect to an increase in free, bioactive curcumin. Without a concurrent measurement of free curcumin, determination of total curcumin by itself gives no indication of the free curcumin proportion, limiting conclusions about the potential efficacy and bioavailability of curcumin.

When plasma samples are hydrolyzed, pharmacokinetic values for curcuminoids from standard unformulated 95% curcumin are in the general range of 0.2–0.3 AUC/mg or lower [26, 27, 30, 33, 36]. It is not always easy to determine if enzymatic hydrolysis was used before curcumin quantification in some studies based on a description of the procedures employed. However, when bioavailability results from these studies involving 95% unformulated curcumin are in the same range of the results of studies with hydrolysis [23, 25], sample hydrolysis is a likely explanation, particularly when results from non-hydrolyzed samples are at least an order of magnitude lower [13, 24].

## Methods

The protocol (ESM-CLN#2018 T01) for this study was approved prospectively by the Bio-Kinetic Clinical Applications Institutional Review Board (Springfield, MO) on March 26, 2018. The study was registered with ClinicalTrials.gov, trial registration number: NCT04103788, September 24, 2019.

Generally healthy human subjects (4 male and 4 female) were recruited into the study based on the inclusion and exclusion selection criteria previously described [13]. The subjects had an average age of 50.6 years and a body mass index (BMI, kg/m^2^) of 30.3. The sample size was adequate for this proof-of-concept study, aiming to test the impact of the enzymatic hydrolysis step on the plasma curcumin profile. No attempt was made to assess effects of age, gender or BMI relative to the effects of enzymatic hydrolysis of the plasma samples. All subjects reviewed and signed an informed consent form. Following consumption of a standard FDA high-fat diet, each subject orally consumed with 240 mL of water a micellar advanced absorption study product containing 400 mg curcumin, coconut oil, polysorbate-20, and DL-alpha-tocopherol which was delivered in six number zero (#0) vegetarian capsules.

Blood samples were collected in vacutainer tubes with EDTA as the anticoagulant from each subject over a 6 h time period (0, 0.5, 1.0, 1.5, 2.0, 4.0, and 6.0 h). The samples were immediately centrifuged at 2500 g at 4 °C for 15 min, and aliquots of 1 mL plasma supernatant were collected and deposited into cryotubes which were immediately frozen and stored at − 80 °C until analyzed [13].

Aliquots of each plasma sample were treated with glucuronidase and sulfatase enzymes as described by Asai and Miyazawa [41]. All enzymatically hydrolyzed and non-hydrolyzed plasma samples were extracted according to the method of Cao et al. [42]. The samples were analyzed for free curcumin, curcumin sulfate, curcumin glucuronide, tetrahydrocurcumin, demethoxycurcumin and bis-demethoxycurcumin by high performance liquid chromatography/tandem mass spectrometry (HPLC/MS/MS) as previously described [13]. Area-under-the-curve (AUC) and C_max_ values were determined using GraphPad Prism 7.0c. Free curcumin equivalents based on molecular weights of each form (free curcumin being 100%) were calculated to derive the actual free curcumin quantities (corrected) for each analyte.

## Results

Direct extraction of plasma samples results in the detection and determination of free curcumin, free demethoxycurcumin, free bis-demethoxycurcumin, curcumin glucuronide and curcumin sulfate. Following enzymatic hydrolysis, curcumin, tetrahydrocurcumin, demethoxycurcumin and bis-demethoxycurcumin can be detected in measurable amounts. Small amounts of demethoxycurcumin and bis-demethoxycurcumin are naturally present in the curcumin product that was administered.

The effects of enzymatic hydrolysis on the AUC and C_max_ data of the primary curcuminoids in plasma of human subjects are presented in Tables 1 and 2, respectively. The results indicate that enzymatic hydrolysis increases the amount of total curcuminoids detected in plasma by a factor of approximately 31-fold. Curcumin glucuronide constituted the primary curcuminoid metabolite detected in non-hydrolyzed plasma. The amounts of curcumin sulfate, demethoxycurcumin and bis-demethoxycurcumin were near the limits of detection in non-hydrolyzed samples, while demethoxycurcumin and bis-demethoxycurcumin were not detected in hydrolyzed samples, therefore no data are presented for these metabolites. No tetrahydrocurcumin was detected in non-hydrolyzed plasma, while it constituted the majority of total curcuminoids detected in plasma that has been hydrolyzed (Table 1). No tetrahydrocurcumin glucuronide standard was commercially available for the determination of this metabolite in non-hydrolyzed samples. These results strongly suggest that curcumin was reduced to tetrahydrocurcumin and then rapidly conjugated. When the amounts of curcuminoids in non-hydrolyzed plasma are corrected with the amount of tetrahydrocurcumin as determined by hydrolysis, it apparent that the total amounts of curcuminoids are similar with and without hydrolysis.

**Table 1:** AUC_0-6h_ ± SEM (ng*h/mL) for human plasma samples from 8 subjects that were assayed with and without enzymatic hydrolysis

NON-HYDROLYZED			HYDROLYZED		
Curcumin	Curcumin Glucuronide	Total Curcumin^a^	Curcumin	Tetrahydrocurcumin	Total Curcumin^a^
0.36 ± 0.71	78.2 ± 20.3	53.9 ± 13.9	486 ± 105	1449 ± 217	1676 ± 179

^a^Corrected—curcumin quantity from each form adjusted by molecular weights for each analyte

**Table 2:** C_max_ ± SEM (ng/mL) for human plasma samples from 8 subjects that were assayed with and without enzymatic hydrolysis

NON-HYDROLYZED			HYDROLYZED		
Curcumin	Curcumin Glucuronide	Total Curcumin^a^	Curcumin	Tetrahydrocurcumin	Total Curcumin^a^
0.71 ± 0.71	28.8 ± 13.4	19.5 ± 9.0	129.4 ± 44.7	313.5 ± 45.0	369.1 ± 39.1

^a^Corrected—curcumin quantity from each form adjusted by molecular weights for each analyte

The C_max_ value for total curcuminoids increased by a factor of approximately 19-fold when the plasma samples were enzymatically hydrolyzed which freed conjugated curcumin and its metabolites from their sulfate and glucuronide conjugates (Table 2).

## Discussion

The results clearly demonstrate that the use of enzymatic hydrolysis of plasma samples prior to extraction and analysis greatly exaggerates the amount of curcumin detected. As noted above, pharmacokinetic studies with almost all enhanced absorption formulations have used enzymatic hydrolysis to free conjugated curcumin prior to analysis, and then reported the results as “curcumin”. Readers of these studies may not understand that the term “curcumin” or “total curcumin” actually refers almost entirely to inactive curcumin conjugates, and not to free curcumin itself. What is not known is to what extent hydrolysis of plasma samples reflects the amount of free curcumin in plasma as compared to the total amount that is detected (free plus conjugated form). It would be accurate to state results of hydrolyzed plasma samples as free plus conjugated curcumin, not merely curcumin.

A preliminary study [43] as well as a study [13] that used direct sample extraction without hydrolysis and compared the results with pharmacokinetic studies that used enzymatic hydrolysis strongly suggested that enzymatic hydrolysis of plasma samples prior to solvent extraction represented a large over-estimation of the amount of free, bioactive curcumin.

The current study provides information regarding the extent of this over-estimation of curcumin. Use of enzymatic hydrolysis results in an exaggeration of the biological and therapeutic potential of the various enhanced absorption formulations that have relied on hydrolysis for their pharmacokinetic determinations of curcumin. Conjugates of curcumin exhibit minimal representation as biomarkers of therapeutic activity, and cannot be compared to the actions of free curcumin.

Although the use of enzymatic hydrolysis prior to solvent extraction may be useful in comparing absorption of curcumin with other studies that also use the same quantification methods, no information is provided with respect to the free, bioactive curcumin. Enzymatic hydrolysis of plasma samples does not provide a true and accurate reflection of the plasma levels of free, bioactive curcumin. Because a substance is absorbed and present in some form in the blood does not provide an indication of bioavailability, bio-efficacy or imply that it has high therapeutic potential.

The ability of any drug or exogenously administered chemical (xenobiotic) to exert a specific physiological/pharmacological effect will depend upon its absorption, distribution, metabolism and excretion. Phase II biotransformation pathways involved in xenobiotic metabolism, including glucuronidation as well as sulfation, are responsible for the inactivation, enhanced water solubility and subsequent clearance of a wide range of drugs and chemicals from the body, including curcumin and other polyphenols [14–20, 37, 40, 44]. UDP-Glucuronosyltransferases (UGTs) are the primary group of enzymes that catalyze the conjugation of glucuronic acid to polar groups on xenobiotics, resulting in more polar, water-soluble and generally less physiologically active metabolites [44]. These conjugates are also better substrates for ATP-binding cassette (ABC) transporters which facilitate export or efflux of drugs and toxins out of cells and tissues [45, 46].

For the purpose of understanding metabolic pathways and overall metabolism of xenobiotics including polyphenolics such as curcumin, hydrolysis with glucuronidase and sulfatase enzymes may be used [47]. Furthermore, the application of enzymatic hydrolysis is useful when authentic reference standards of the conjugates are not available. However, this is not the case with curcumin because curcumin glucuronide and curcumin sulfate reference standards are available. Furthermore, enzymatic hydrolysis as a reliable analytical procedure in the quantification of glucuronidated and sulfated polyphenolic metabolites has been questioned, and has been shown to negatively affect recovery of the free-forms of polyphenols present in plasma [48]. The study described above provides evidence supporting this conclusion. As a consequence, the data provided by the enzymatic hydrolysis of plasma samples containing curcumin derivatives and metabolites is not a reliable indicator of the actual levels of bio-active curcumin.

Santos et al. [49] reviewed the methods used in studying the pharmacokinetics of polyphenols which includes curcumin. These authors noted that until about the mid-1990s, hydrolysis of biological samples such as plasma was widely used and total aglycones were measured. However, the subsequent development of more sensitive analytical methods as high pressure liquid chromatography coupled with tandem mass spectrometry (HPLC/MS/MS) has enabled the determination of the parent polyphenols and their metabolites including conjugated forms following direct extraction with a high degree of sensitivity and accuracy [47]. Thus, studies involving curcumin pharmacokinetics that utilize hydrolysis of biological samples are not employing the most accurate, reproducible and appropriate techniques.

Based on these considerations, the pharmaceutical pharmacokinetics model and standard protocol involves the determination of blood plasma levels of the active form(s) of a xenobiotic, and not the amounts of the drug that may be conjugated and converted to pharmacologically inactive forms [39, 40]. Tamoxifen is a drug widely used for the treatment of breast cancer, and is an excellent example that can be used to highlight drug pharmacokinetics [50]. Tamoxifen, like curcumin, undergoes metabolism including demethylation, hydroxylation, and extensive glucuronidation [50]. The glucuronides are excreted in the bile and urine, similar to curcumin glucuronide [13], and also undergo urinary excretion. Glucuronidation plays a major role in therapeutic resistance to tamoxifen and inter-individual variability in responsiveness [50]. It is the plasma levels of tamoxifen and its hydroxylated metabolites and not the amount of the glucuronides or a combination of the free and conjugated forms that is indicative of therapeutic potential and efficacy [50]. A similar case can be made for curcumin.

Hydrolysis of plasma samples to assess the pharmacokinetics of tamoxifen or any other xenobiotic including curcumin that undergoes conjugation will result in false and misleading results. Hydrolysis of plasma samples can provide information regarding the conjugated metabolites of any given substance including curcumin, but it does not provide information regarding the amount of free, bioactive curcumin.

## Conclusions

The results of this study show that the use of enzymatic hydrolysis of plasma samples greatly exaggerates the amount of curcumin detected, resulting in a determination of free plus conjugated forms. The majority of pharmacokinetic studies with various formulations designed to enhance curcumin bio-accessibility and bioavailability have not measured free, bioactive curcumin. These studies have reported total (free plus conjugated) curcumin in the plasma after conversion of inactive conjugated forms of curcumin to free curcumin through enzymatic hydrolysis. Free curcumin levels cannot be quantified when enzymatic hydrolysis is employed. Thus, what is reported is not a clear and accurate indication of enhanced plasma levels of free bioactive curcumin as a result of the product formulation, and constitutes a misrepresentation of the results when plasma is enzymatically hydrolyzed. Using enzymatic hydrolysis of plasma samples to free conjugated and inactive forms of xenobiotics to assess bioavailability of bioactive constituents is not an accepted or reliable practice within the pharmaceutical industry, nor should it be an accepted practice within the dietary supplement industry. Based on current knowledge of the biological effects of curcumin and its metabolites, free curcumin in the blood (plasma) is the best indicator of bioavailability and bioequivalence, and therefore this is the indicator that should be measured.

## Data Availability

The datasets used and analyzed during the current study are available from the corresponding author on reasonable request.

## Abbreviations

AUC: Area under the curve
BMI, kg/m^2^: Body mass index
C_max_: Maximum concentration
HPLC/MS/MS: Chromatography/tandem mass spectrometry
t_max_: Time of maximum concentration
